# Different functional networks underlying human walking with pulling force fields acting in forward or backward directions

**DOI:** 10.1038/s41598-023-29231-6

**Published:** 2023-02-02

**Authors:** Tetsuya Ogawa, Hiroki Obata, Hikaru Yokoyama, Noritaka Kawashima, Kimitaka Nakazawa

**Affiliations:** 1 Department of Clothing, Faculty of Human Sciences and Design, Japan Women’s University, Tokyo, 112-8681 Japan; 2 Graduate School of Arts and Sciences, The University of Tokyo, Tokyo, 153-8902 Japan; 3 School of Engineering, Kyushu Institute of Technology, Kita-Kyushu, 804-8550 Japan; 4 National Rehabilitation Center for Persons with Disabilities, Saitama, 359-8555 Japan

**Keywords:** Central pattern generators, Cerebellum, Spinal cord

## Abstract

Walking with pulling force fields acting at the body center of mass (in the forward or backward directions) is compatible with inclined walking and is used in clinical practice for gait training. From the perspective of known differences in the motor strategies that underlie walking with the respective force fields, the present study elucidated whether the adaptation acquired by walking on a split-belt treadmill with either one of the force fields affects subsequent walking in a force field in the opposite directions. Walking with the force field induced an adaptive and de-adaptive behavior of the subjects, with the aspect evident in the braking and propulsive impulses of the ground reaction force (difference in the peak value between the left and right sides for each stride cycle) as parameters. In the parameters, the adaptation acquired during walking with a force field acting in one direction was transferred to that in the opposite direction only partially. Furthermore, the adaptation that occurred while walking in a force field in one direction was rarely washed out by subsequent walking in a force field in the opposite direction and thus was maintained independently of the other. These results demonstrated possible independence in the neural functional networks capable of controlling walking in each movement task with an opposing force field.

## Introduction

Despite its stereotypical features that exhibit stable rhythmicity and reproducibility, human locomotion is flexible enough to meet changing task demands. From the perspective of its kinematics, the lower limb joints exhibit similarity to repeat flexion and extension over gait cycles, regardless of demand. However, recent studies have shown the possibility of different neural mechanisms (or motor strategies) underlying locomotion that is dependent on detailed tasks^1–6^. Based on the locomotor adaptation that occurs in a particular locomotive task, the occurrence of aftereffects in other locomotive tasks or contexts was less evident^1–6^. These studies particularly focused on slow component of locomotor adaptation that occurs over minutes as a consequence of comparison between predicted and actual limb movement and the recalibration of motor output through cerebellar function^7^ upon exposure to a novel mechanical environment. These studies suggest the possibility of different neural networks responsible for each locomotive task (i.e., direction, speed, gait mode [walk and run], and use of hand-held poles). Contrary, several studies suggest the use of common neural network among different locomotor tasks on the basis of analysis of limb kinematics^8–10^ and electromyographic (EMG) activities in locomotor-related lower limb muscles^8,10^. Provided that humans locomote in a variety of environments (such as leveled ground, slopes, slippery floor) by utilizing different tasks (such as walking and running, or those under different speed) depending on demand, it is particularly important to understand the responsible control mechanisms.

To further investigate the basic features of the neural mechanisms underlying human locomotion and particularly from a perspective of motor adaptation, the present study focused on walking with force fields acting at the body center of mass (COM), which pull subjects forward (aiding force field) or backward (impeding force field). Applying these force fields to normal unperturbed walking has been shown to alter the mechanical demands associated with braking and propulsion. Aiding force fields are known to increase braking and reduce propulsive impulses, while the impeding force field increases propulsive but reduces braking impulses^11^. Recently, it has been demonstrated that an application of horizontal impeding force affects metabolic power in running gait and increases 6.13% per 1% body weight of horizontal impeding force^12^. Along with the changing mechanical demands, the application of these force fields reduced (aiding force field) and increased (impeding force field) the effort to walk and the activity of the plantar flexor muscle (medial gastrocnemius), which is necessary for propulsion^11,13^. Furthermore, a recent study demonstrated detailed characteristics in the adjustment of walking with these force fields, including different COM dynamics and spinal motor output^14^. In this study, the analysis of EMG activity in 16 muscles demonstrated not only a general increase or decrease in activity, but also specific changes dependent on muscles and the direction of the force fields, particularly in terms of activity in the lumbar and sacral motor pools of the spinal cord. Importantly, the results of the force fields acting on the COM resemble those obtained when walking on a slope^15^ resulting in modifications of positive and negative COM mechanical power production^14^ and the strategies of neuromuscular control^16,17^. To add, similarity in the mechanical adjustments of locomotion with the application of force fields to those performed on slope have been performed in running, recently^18^. Following its compatibility with inclined walking in our daily lives along with its possible use in the clinical practice of gait training^19^, a detailed analysis of walking with these force fields is expected to play a crucial role in further understanding gait control.

The present study utilized locomotor adaptation on a split-belt treadmill, a novel environment in which two belts (one underneath each foot) were driven at different velocities to another. Known that the aspect of adaptation thorough walking on the split-belt treadmill is evident in the interlimb adjustment of gait, particularly in the kinetic adjustments between that limb^20^, rather than intralimb^21^, the present study focused on the asymmetry of the ground reaction force between the two limbs^3–6^ and addressed whether the adaptation was transferred between walking with different force fields. With the known task-dependent specificity in the neural mechanisms underlying human locomotion as demonstrated in a series of motor adaptation studies^1–6^, along with difference in COM dynamics and spinal motor output^14^ it was possible that further specificity underlies between walking in the specific mechanical environment with the pulling force field into opposite directions. It was hypothesized that adaptation transfers only limitedly and is maintained independently between walking conditions with pulling force filed acting into opposite directions as a reflection of specific neural mechanisms being capable of walking in the given environment. That is, after adaptation to walk on an asymmetrically-driven split-blet treadmill under pulling force filed in one direction, aftereffect is evident as asymmetry of the ground reaction force between the two limbs when subsequently walking on a symmetrically-driven treadmill under force filed into the same direction, but less evident under force field into opposite direction. Further, aftereffect (asymmetry of the ground reaction force between the two limbs) acquired through walking on an asymmetrically-driven split-blet treadmill under pulling force filed in one direction is washed out (or decays) through subsequent walking on a symmetrically-driven treadmill under force filed into same direction, but less affected by walking under force field into opposite direction.

## Materials and methods

### Participant

**Figure 1:**
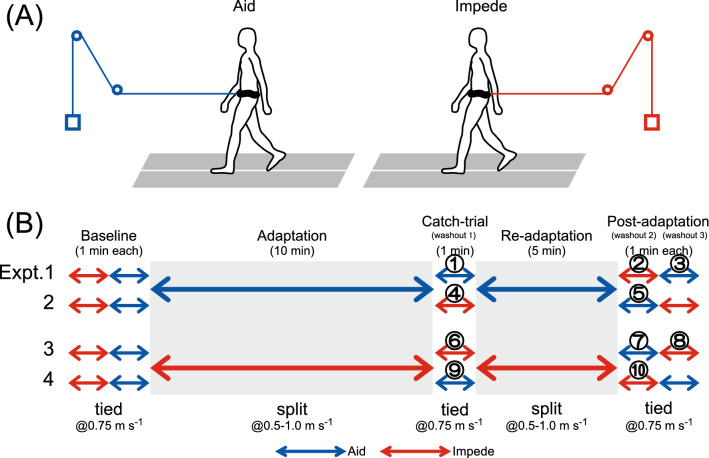
(**A**) Experimental apparatus used to impose aiding (left) and impeding (right) force fields on subjects. The belt stranded around the torso near the center of mass (COM) was attached to the weight (2 kg) by a cable and two low friction pulleys. (**B**) Experimental protocols used in the present study. In Experiments 1 and 2, subjects underwent a split-belt adaptation with aiding force field and in Experiments 3 and 4, with impeding force field.

Sixteen volunteers (15 males and 1 female; mean ± SD age, 28.6 ± 6.6 years; height, 172.0 ± 7.7 cm; body weight (BW), 66.0 ± 12.0 kg) without a history of neurological or orthopedic disorders were included in this study. Each participant was tested using two of the four experimental protocols (Fig. 1B). Eight of them participated in Experiments 1 and 3, while the other eight participated in Experiments 2 and 4, with the order of participation randomly distributed among subjects to overcome any ordering effects. All participants provided written informed consent before participation. All experimental procedures were approved by the local Ethics Committee of the School of Arts and Sciences of the University of Tokyo and were conducted following the Declaration of Helsinki.

### Experiment

The experiments consisted of walking under one of two physical conditions (either with an “aiding” the force field or an “impeding” force field, see Fig. 1A). Force fields were applied to the participants via a belt stranded around the torso near the COM, which was then attached to the counterweight (2 kg, corresponding for 3.12 ± 0.54% BW of the subjects) through two carabiners, a rigid cable, and two low friction pulleys. Subjects were pulled horizontally forward in the aiding force field, while a backward pull was applied in the impeding force field.

The participants were instructed to walk on a split-belt treadmill (Bertec, Columbus, OH, USA) with two separate belts, and the speed of each belt was controlled independently. In the present experiment, the treadmill was operated under one of two conditions, tied (two belts moving together at the same speed) or split (separately at different speeds), using a custom-written computer program written in Lab-VIEW (National Instruments, Austin, Texas, USA). The speeds were set at 0.75 ms^−1^ for both belts under the tied, and while in the split, the belt on the left side was 0.5 ms^−1^ and another on the right was 1.0 ms^−1^ (ratio, 1:2). The limb on the slower (left) side speed of the treadmill under the split was defined as the “slow limb” and the limb on the faster (right) side speed as the “fast limb”.

The experimental protocols consisted of baseline, adaptation, re-adaptation, and three washout periods, as dictated by the protocol (Fig. 1B). Participants always accompanied one of the two force fields (impeding or aiding) throughout the experiments. During the baseline, the treadmill was tied and participants walked with the impeding and aiding force fields for 1 min each. The treadmill was then operated in a split and the participants underwent a 10-min adaptation, followed by a 1-min catch trial (washout 1) to walk on the treadmill in tied. The treadmill was then returned to split, and the participants again underwent adaptation to walking on a split-belt (re-adaptation) for 5 min, which was again followed by a 1-min catch trial (washout 2) to walk on a tied belt. The force fields in the two catch trial periods (washouts 1 and 2) were different in direction (impeding washout 1 and aiding in washout 2 or vice versa) to address both the degree of adaptation by evaluating the magnitude of the aftereffect and how it could transfer to walking with the opposite force field. In Experiment 1, for example, the degree of adaptation was tested by assessing the magnitude of the aftereffect while walking with the aiding force field on the tied belt during washout 1 (catch trial) after adapting to walk on the split-belt with the force field in the same (aiding) direction. The transfer of adaptation, on the other hand, was tested by walking with the impeding force field in washout 2 (post-adaptation) after adapting to walking (re-adaptation period) with the force field in the opposite (aiding) direction. Given that the emergence of the aftereffect is not stable but can decay throughout the experiments^4,22^, the order of exposure to the washout periods with different force fields was alternated depending on the experiments (between experiments 1 and 2, 3, and 4, respectively) to overcome possible ordering effects.

Subjects underwent an extra washout period (washout 3, ➂ and ➇ in the experiments 1 and 3, respectively and those in the experiments 2 and 4 not subject for further analysis) to walk on a tied belt with force fields in the same direction as the adaptation period, but were different from those during the washout 2 period. The purpose of this additional washout period was to evaluate the degree to which the adaptation acquired through walking on the split-belt could be maintained (or washed out) after walking with a force field in a different direction. Between each testing period, upon changing the belt speeds and/or direction of the force field, there was a 15-s time interval in which subjects stepped on platforms on both sides of the treadmill. They were then allowed to step on the treadmill with the left leg when a sufficient belt speed was reached and the appropriate force fields were mounted. During the experiments, subjects were instructed to walk while watching a wall approximately 3 m in front of them and not to look down at the belts. They were allowed to hold onto the handrails mounted on either side of the treadmill in case of risk of falling. All subjects completed the test sessions without holding on. To ensure safety, one experimenter stood by the treadmill.

### Data recording and analysis

Force sensors mounted underneath each treadmill belt were used to determine the dimensional ground reaction force (GRF) components: mediolateral (Fx), anteroposterior (Fy), and vertical (Fz). Force signals were sampled at 1 kHz, stored on a computer via an analog-to-digital converter, and low-pass filtered at a cut-off frequency of 8 Hz (Power Lab; AD Instruments, Sydney, Australia). The anteroposterior (Fy) and vertical (Fz) components were used in the later off-line analysis. The magnitude of the anteroposterior (Fy) GRF component was evaluated for each stride cycle. The timing of foot contact and toe-off for each stride cycle was determined based on the vertical Fz component of the GRF for both fast and slow sides using custom-written software (VEE Pro 9.3, Agilent Technologies, Santa Clara, CA, USA).

**Figure 2:**
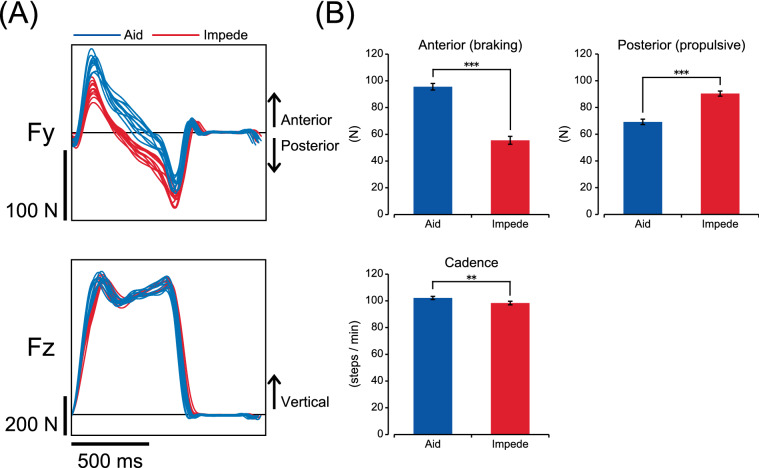
(**A**) Representative examples of the anteroposterior GRF (N) and the vertical GRF (N) during baseline with exposure to different force fields. Each set of waveforms represents the time-series changes of the force for ten consecutive stride cycles (from heel contact to subsequent heel contact and including both left and right sides, superimposed) in a single subject. The blue and red lines represent walking with an aiding force field and an impeding force field, respectively. The calibration bars indicate 100 N (Fy), and 200 N (Fz) for the vertical axis and 500 ms for the horizontal axis, respectively. (**B**) Mean amplitude (absolute) of each GRF component tested during the baseline with different force fields. The error bars represent the SEM. Statistically significant differences: **P < 0.01, ***P < 0.001, n = 16.

**Figure 3:**
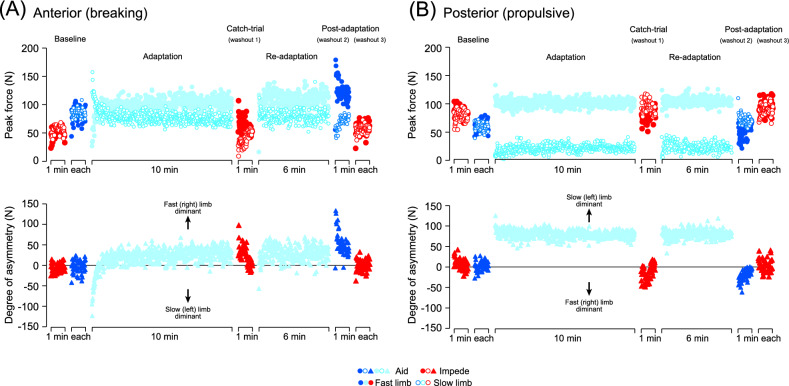
(Upper panels) Example of typical time-series changes in the peak amplitude (absolute values) of the braking (**A**) and the propulsive (**B**) component of the GRF on a stride-by-stride basis in a single subject (same subject as in Fig. 4) from Experiment 2 (split-belt adaptation with aiding force field) for both the fast (filled circle) and slow (open circle) sides. (Lower panels) The differences in the peak forces between the fast and slow sides (degree of asymmetry) for each stride cycle.

**Figure 4:**
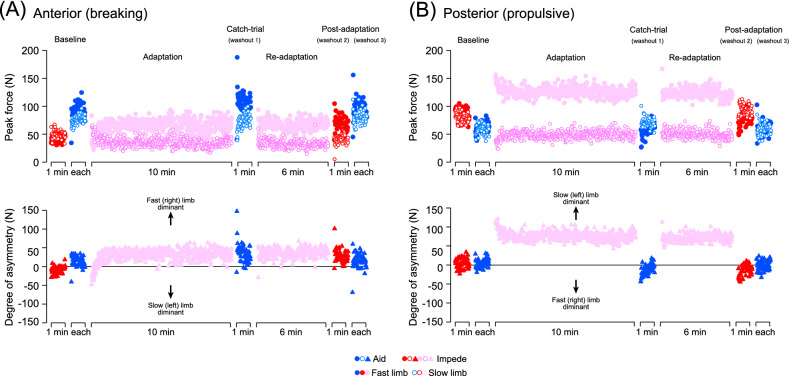
(Upper panels) Example of typical time-series changes in the peak amplitude (absolute values) of the braking (**A**) and the propulsive (**B**) component of the GRF on a stride-by-stride basis in a single subject (same subject as in Fig. 3) from Experiment 4 (split-belt adaptation with impeding force field) for both the fast (filled circle) and slow (open circle) sides. (Lower panels) The differences in the peak forces between the fast and slow sides (degree of asymmetry) for each stride cycle.

To address the degree of adaptation and transfer of motor patterns across walking with force fields in opposite directions, the degree of asymmetry in the anteroposterior (Fy) component of the GRF was calculated for each stride cycle of walking. As depicted in Fig. 2A, this GRF component includes braking and propulsive components that appear at different phases during the gait cycle. For each component, the peak amplitude during each gait cycle was calculated as the absolute value for both the fast and slow sides (upper panels of Figs. 3, 4). The degree of asymmetry, which represents the difference in the absolute values, was then calculated by subtracting the value of the slow limb from that of the fast limb on a stride-by-stride basis (lower panels of Figs. 3, 4).

As exposure to different force fields (aiding and impeding) influences the magnitude of the GRF components during walking, it does not allow for direct comparisons of the degree of asymmetry between walking with different force fields. In addition, to consider the influence of the natural walking movement of the subjects, which is not perfectly symmetrical and allows for comparisons between different force fields, the obtained degree of asymmetry underwent a normalization process. For both the braking and propulsive components of the GRF, the degree of asymmetry obtained in the washout periods (1, 2 and 3) was subtracted by the mean values of those under the respective baseline with different force fields. The normalized values were then divided into bins of 5 s and averaged for each bin.

### Statistics

Two-way analysis of variance (ANOVA) with repeated measures was used to test for statistically significant differences in the degree of asymmetry (in terms of both acquisition and transfer of adaptation) between walking with different force fields (either aiding or impeding) and different time periods of the experiment (initial or final phase of washout periods). When ANOVA revealed significant results, Bonferroni’s post hoc comparisons were performed to identify significant differences between variables. In addition, to test whether the adaptation acquired during walking with one force field was washed out (or maintained) by walking with the other force field, a paired Student’s t-test was performed to compare the degree of asymmetry between the final phase of washout 2 and the initial phase of washout 3 periods. A paired Student’s t-test was used to compare the magnitude of the GRF components and the cadence between walking under the two force conditions. Data are presented as mean ± standard error of the mean (SEM) values. Statistical significance was set at P < 0.05.

## Results

The addition of both aiding and impeding force fields during walking resulted in a systematic modification of the magnitude of the GRF components. Figure 2A,B shows the changes in each GRF component depending on the force field while walking normally on the tied belt during the baseline. Figure 2A shows the typical GRF waveforms for 10 consecutive stride cycles (heel contact to heel contact, superimposed) under each force field in a single subject, and Fig. 2B shows the group means of the peak magnitude of each GRF component for each stride cycle as well as the cadence. The anteroposterior Fy component includes the braking component and the propulsive component. With the aiding force field, the braking component was significantly greater than with the impeding force field (P < 0.001). In contrast, the propulsive component was significantly larger with the impeding force field than with the aiding force field (P < 0.001). There was also a statistically significant difference in cadence (P < 0.01), where it was slightly higher with aid (102.2 ± 1.2 steps/min) than with the impeding force field (98.3 ± 1.3 steps/min).

Figures 3 and 4 show representative examples of time-series changes in the braking (A) and propulsive component (B) of the GRF throughout the experiments on a single subject. In both figures, the upper panels show the peak amplitude of the GRF components on a step-by-step basis for each limb (left to right), whereas the lower panels show the difference in the peak amplitude between the limbs (degree of asymmetry) for each stride cycle. During the baseline period, both the braking and propulsive components showed similar amplitudes between the sides with certain variability between each stride cycle, regardless of whether the force field was aiding or impeding (upper panels of Figs. 3, 4). Consequently, the values of the degree of asymmetry (lower panels of Figs. 3, 4) for each stride cycle are scattered around zero (horizontal lines indicate perfect symmetry). With exposure to the split-belt, subjects exhibited a pronounced limp in their walking pattern in the early phases, followed by slower changes to walk more stably. These modifications in walking patterns are quantified as changes in the GRF in both the braking and propulsive components, in which the braking component generally showed more prominent changes than the propulsive component. With a return to the tied belt in the catch trial and post-adaptation periods, significant “aftereffects” (hereafter, used to show fast (right) limb–slow (left) limb asymmetry of the GRF) with the degree of asymmetry deviating in the opposite direction to those during the early phases of adaptation and re-adaptation periods were observed. In particular, a significant after effect was observed in the braking and propulsive components, which showed only minor effects in previous studies of split-belt adaptation without additional force fields^19,21^.

**Figure 5:**
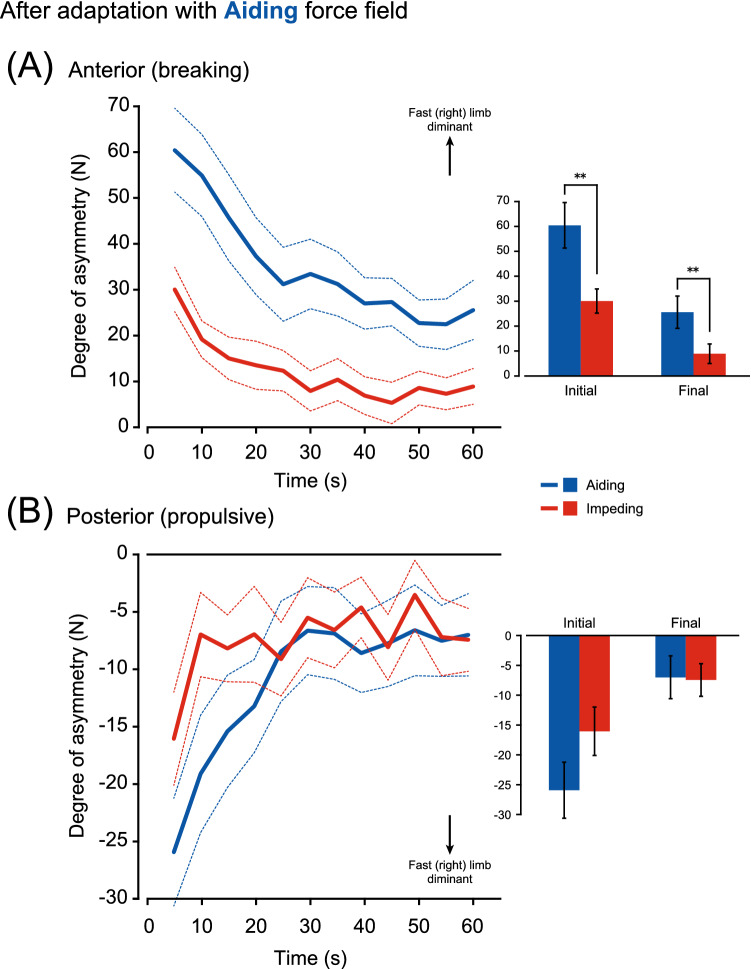
Comparison of the after effect sizes in the braking component (**A**) and the propulsive component (**B**) of the GRF between walking with aiding (blue lines) and impeding (red lines) force fields after adaptation with the aiding force field. Over the 1 min washout period, data were averaged for every 5-s bin. Data of eight subjects were obtained during washout 1 (corresponding to ➀ or ➃ in Fig. 1B), while those of the other eight subjects were from washout 2 (➁ or ➄) to overcome the ordering effects (n = 16). Lines show the mean (solid) and standard error of the mean (dotted), respectively. The bar graphs highlight the initial and final 5 s of the 1 min washout period. The error bars are the standard error of the mean. **P < 0.01.

**Figure 6:**
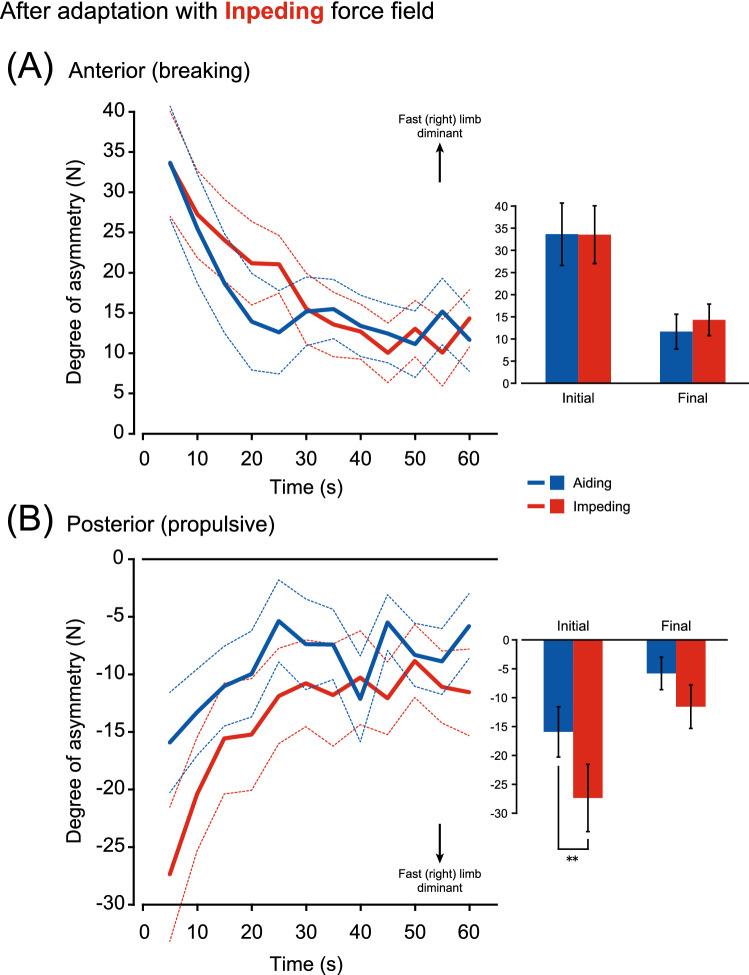
Comparison of the after effect sizes in the braking component (**A**) and the propulsive component (**B**) of the GRF between walking with aiding (blue lines) and impeding (red lines) force fields after adaptation to the impeding force field. Over the 1 min washout period, data were averaged for every 5-s bin. Data of eight subjects were obtained during washout 1 (corresponding to ➅ or ➈ in Fig. 1B), while those of the other eight subjects were from washout 2 (➆ or ➉) to overcome the ordering effects (n = 16). Lines show the mean (solid) and standard error of the mean (dotted), respectively. The bar graphs highlight the initial and final 5 s of the 1 min washout period. The error bars are the standard error of the mean. **P < 0.01.

In Figs. 5 and 6, the extent of the transfer of adaptation between walking with different force fields is portrayed by comparing the degree of asymmetry between walking with an aiding force field and that with an impeding force field during the catch trial and washout periods. A significant aftereffect was evident, regardless of the GRF component (braking or propulsive). However, the magnitude of the aftereffect was largely dependent on the component and the combination of the force field between the adaptation and catch trial/washout periods. In the braking component after adaptation to the aiding force field (Fig. 5A), ANOVA revealed that there were significant main effects for the type of force field (aiding or impeding) (F(1,15) = 57.91, P < 0.001) and time (initial or final epoch of the catch trial and washout periods) (F(1,15) = 27.20, P < 0.001) and significant interaction (F(1,15) = 5.98, P < 0.05). The aftereffect was greater when walking with an aiding force field than with an impeding one in the initial (P < 0.01) and final (P < 0.01) epochs of the catch trial/washout periods. However, there were significant differences in the propulsive component (Fig. 5B) depending on the time (F(1,15) = 33.23, P < 0.001) but not on the type of force field (F(1,15) = 1.08, P = 0.31). This interaction was also significant (F(1,15) = 5.93, P < 0.05).

In comparison of aftereffects after adaptation to the impeding force field (Fig. 6), differences dependent on the type of force field were only evident in the propulsive component (Fig. 6B) and not in the braking component (Fig. 6A). There was a difference in the braking component depending on the time (F(1,15) = 16.24, P < 0.001), but not on the type of force field (F(1,15) = 0.14, P = 0.72). This interaction was not significant (F(1,15) = 0.22, P = 0.65). In contrast, in the propulsive component (Fig. 6B), there was a significant difference in the type of force field (F(1,15) = 11.07, P < 0.01) and time (F(1,15) = 10.44, P < 0.01). This interaction was not significant (F(1,15) = 1.16, P = 0.30). The size of the aftereffect was greater while walking with an impeding force field than with aiding in the initial phase of the catch trial/washout period (P < 0.05).

**Figure 7:**
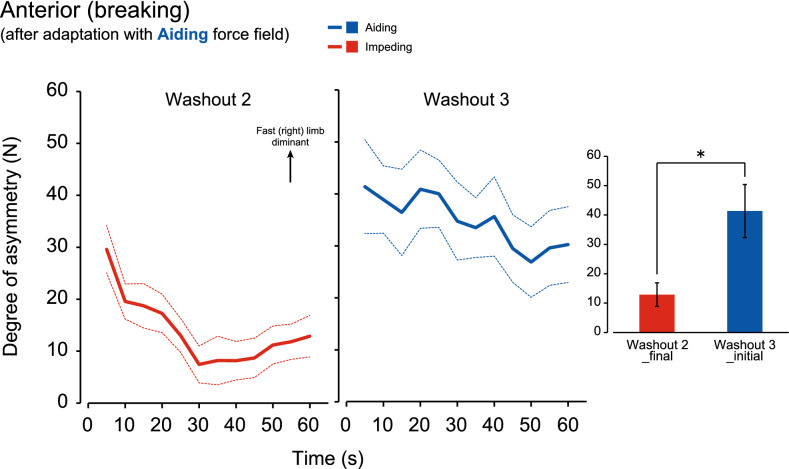
Degree of washout in the acquired asymmetrical movement pattern in the braking GRF component with the aiding force field by subsequent walking with the impeding force field in washout 2 (➁) and the aiding force field in washout 3 (➂) periods (n = 8). The lines represent the mean (solid) and the standard error of the mean (dotted). The bar graph compares the mean values between the last 5 s bin of washout 2 (➁) and the first 5 s bin of washout 3 (➂). The error bars represent the standard error of the mean. *P < 0.05.

**Figure 8:**
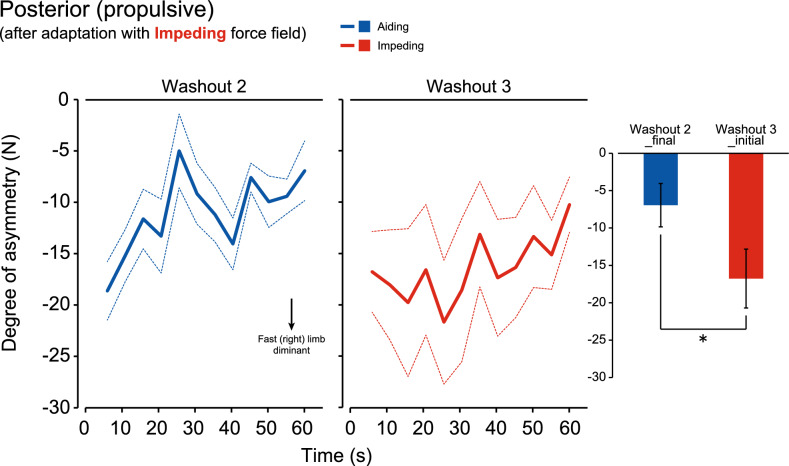
Degree of washout in the acquired asymmetrical movement pattern in the propulsive GRF component with the impeding force field by subsequent walking with the aiding force field in washout 2 (➆) and impeding force field in washout 3 (➇) periods (n = 8). The lines represent the mean (solid) and the standard error of the mean (dotted). The bar graph compares the mean values between the last 5 s bin of washout 2 (➆) and the first 5 s bin of washout 3 (➇). The error bars represent the standard error of the mean. *P < 0.05.

Provided that the transfer of adaptation occurred only partially in the particular combination of the force field and the GRF component (Figs. 5A, 6B), the extent of washout in the adaptation was further investigated. Figures 7 and 8 show the group means of the degree of asymmetry for washout period 2 and subsequent washout period 3 among subjects who underwent Experiments 1 and 3, respectively. As shown in Fig. 5A, the motor pattern acquired through walking with an aiding force field was partially transferred to subsequent walking with an impeding force field, as observed in the braking GRF component. A similar tendency is shown in Fig. 7 (red line) as a group means of eight subjects, where the degree of asymmetry deviates in the positive direction and decays in the subsequent 60 s washout period. Once the direction of the force field was switched from impeding to aiding, the degree of asymmetry increased significantly (blue line). There was a significant difference in the degree of asymmetry between the final epoch of the second washout period and the initial epoch of the third washout period (bar graph, P < 0.05). In the result of the propulsive GRF component after adaptation to the impeding force field (Fig. 8), the degree of asymmetry showed a large increase upon a change in the direction of the force field from aiding to impeding between washout periods 2 and 3. There was a significant difference in the degree of asymmetry between the final epoch of the second washout period and the initial epoch of the third washout period (bar graph, P < 0.05).

## Discussion

The present study investigated whether the adaptation of walking with force fields acting on the body COM of subjects in one direction (either forward or backward by aiding and impeding force fields, respectively) transfers to another in the opposite direction. Subjects walked on a split-belt treadmill where two belts were driven at different speed to each other (one belt at 0.5 ms^−1^ and another at 1.0 ms^−1^ (therefore, 1:2 in ratio). Known that the number of stride cycle needed for adaptation is dependent on the speed ratio of the two belts^21^, subjects underwent adaptation to store novel motor pattern to walk in the constrained environment. With the subjects fully-adapted to walk in the constraints, subsequent change of the belt speeds to normal condition (two belts driven in the same speed) resulted in the emergence of after effect (fast (right) limb − slow (left) limb asymmetry of the GRF). With the braking and propulsive components of the GRF as a parameter, the transfer occurred only limitedly between walking with the opposite force field, with the results dependent on the parameter. When adaptation occurred by walking with an aiding force field, the aftereffect size was greater in walking with an aiding force field than in walking with impeding only in the braking component. The aftereffect was smaller with an impeding force field; however, it became larger when the force field was switched from impeding to aiding, showing limited washout with the opposite force field. Meanwhile, when adaptation was acquired by walking with an impeding force field, the aftereffect was greater in walking with an impeding force field than in walking with aiding force field only in the propulsive component. At this time, the smaller aftereffect of walking with the aiding force field became larger as the direction of the force field switched to impeding. Together, these results support the hypothesis of the present study and suggest that human locomotion with force fields acting in different directions is controlled by different neural circuits in the central nervous system.

The aiding and impeding force field used in the present study was smaller in size (2 kg counterweight accounts for approximately 3% mean body weight of the subjects) compared to those used in previous studies using up to 15% body weight of the subjects^11,14^. However, the application of this relatively smaller force field resulted in constant changes in both the braking and propulsive impulses, along with other GRF components and cadence (Fig. 2). The application of the force field with different directions not only revealed the use of different neural mechanisms dependent on the direction of force fields but also changed the strategies used by the subjects to adapt to walking on a split-belt treadmill (i.e., adaptive and de-adaptive aspects were evident in parameters different from walking without force fields).

In the emergence of the aftereffect, the present study demonstrated results contrasting with our previous ones, in which adaptive processes and subsequent aftereffects were less evident in the propulsive component of the GRF^20,22^. The results showed clear adaptive and de-adaptive aspects only in the braking component associated with activity in the tibialis anterior muscle during the early stance phase, suggesting that predictive feedforward control was required to set the optimal ankle stiffness upon ground contact^20^. This was in contrast to the propulsive component that showed only constant (not adaptive) changes following the fast/slow speed of the split-belt along with activity in the gastrocnemius muscle during the stance phase, showing the use of passive feedback control for the production of reflexively induced propulsive force at the end of the stance phase^20^. Meanwhile, in this study, clear adaptive processes and aftereffects in the propulsive component were evident (as portrayed in the representative example, especially in Fig. 4, and in the mean values in Figs. 5, 6) in addition to the braking component. Given that the predictive feedforward process of the nervous system underlies both adaptive and de-adaptive processes, the present results showed possible changes in the adaptive strategy with the additional involvement of predictive feedforward control to recalibrate the motor output necessary for propulsion in the given environment. The application of the force field while walking reduces (aiding force field) and increases (impeding force field) the effort to walk and muscle activity in the plantar flexor muscle necessary for propulsion^11^. Therefore, emphasizing the propulsion phase with the application of the impeding force field is possible to enhance the use of the predictive feedforward process at the end of the stance; therefore, adaptive and de-adaptive (aftereffect) processes emerged in the propulsive GRF component. Meanwhile, adaptive and de-adaptive processes were also evident in the propulsive GRF component, which was also evident with the application of the aiding force field and is contradictory to the perspective of propulsion effort. It is possible that the adaptation strategy of the subjects to walk on the split-belt may have been affected by the application of the force fields regardless of the direction (aiding or impeding). In the present study focusing on transfer and washout of adaptation between walking with different task demand (application of pulling force field of different direction), it is of particular interest that both transfer and washout took place only limitedly in the specific parameters. The result was evident in the GRF component that were emphasized during the adaptation periods (in the braking component after adaptation with aiding force and in the propulsive component after adaptation with impeding force) but not in others.

What factors were responsible for the partial transfer of washout between walking with different force fields acting on the subjects? Given the possibility of different functional networks depending on detailed locomotion tasks, studies have demonstrated differences in the combination of muscle or neural sites used in different locomotion tasks. For example, in forward and backward walking, the organization of muscle synergies in the activity of lower extremity muscles has been demonstrated to be different^23^. Among the different speeds of walking and running, spinal mapping reconstructed from the activity of 14 lower extremity muscles demonstrated that a different site of the spinal cord was used depending on gait and speed^24^. Differences in the activity of the spinal cord based on spinal mapping were also demonstrated in walking with the application of force fields in the trunk, as in the present study. When subjects walked on a treadmill at speeds of 0.83, 1.39, 1.94 ms^−1^, either one aiding or impeding force, each corresponding to 15% of the body weight of the subjects, was applied at the COM^14^. The results showed that the activity of the sacral motor pool increased with the impeding force and decreased with the aiding force, while in the lumbar motor pool, the activity increased with both aiding and impeding forces. Interestingly, the results were similar to those obtained when walking on a slope. While the activity of the sacral motor pool increased on a positive slope and decreased on a negative slope, in the lumbar motor pool, the activity increased on both positive and negative slopes^15^.

Underlying the differences in the combination of muscles and neural sites used depending on the detailed task of locomotion, differences in cadence cannot be ruled out. In experiments investigating the locomotion of nonhuman animals, the emergence of locomotor behavior concerning the underlying neural mechanisms was dependent on cadence (movement frequency). In the swimming behavior of larval zebrafish, McLean et al.^25^ demonstrated that particular groups of spinal interneurons were active in a particular swimming frequency range and were inhibited and kept silent at other swimming frequencies. In the spinal interneurons responsible for the alternate movement from left to right of the stepping movement in cats, the alternate movement of the limbs was diminished with a specific frequency of movement when a particular set of interneurons underwent lesions^26^. The present results demonstrated a minor but constant difference in cadence between walking with aiding (102.2 ± 1.2 steps min^−1^) and impeding force fields (98.3 ± 1.3 steps min^−1^). These differences could influence the limited transfer and washout of adaptation between walking with different force fields.

As a limitation of the present study, it was possible that the traction force imposed on the subject was not constant through each stride cycle. This is because the present experiment used a rigid cable and it was therefore an issue with inertia. A use of rubber tubing or spring placed in series with the rigid cable along with force transducer was more appropriate for the purpose of the present study.

To summarize, the present results revealed that the adaptation that occurs while walking with a force field acting on the COM of the subjects in one direction does not transfer to walking with a force field in the opposite direction and is rarely washed out by each other. These results demonstrate the independence of neural control of these locomotor tasks and provide basic knowledge to better understand the specificity of the tasks underlying human locomotion. With its compatibility with walking on slopes, the results can provide helpful information to develop intervention strategies for gait training in clinical practice. Given that the training effect was more evident within the specific task or environment of walking and less under others, clinical practice of gait directed towards everyday life, conducted in a variety of environments and by utilizing different tasks should take account for the specificity of adaptation (training effect) dependent on the environment and the task of walking.

## Data Availability

The datasets used and/or analysed during the current study available from the corresponding author on reasonable request.
